# Effect of PACAP in Central and Peripheral Nerve Injuries

**DOI:** 10.3390/ijms13078430

**Published:** 2012-07-06

**Authors:** Andrea Tamas, Dora Reglodi, Orsolya Farkas, Erzsebet Kovesdi, Jozsef Pal, John T. Povlishock, Attila Schwarcz, Endre Czeiter, Zalan Szanto, Tamas Doczi, Andras Buki, Peter Bukovics

**Affiliations:** 1 PTE-MTA “Lendulet” PACAP Research Team, Department of Anatomy, University of Pecs, Szigeti. u. 12, H-7624 Pecs, Hungary; E-Mails: dora.reglodi@aok.pte.hu (D.R.); endre.czeiter@gmail.com (E.C.); 2 MTA-PTE Clinical Neuroscience MR Research Group, Department of Neurosurgery, University of Pecs, Ret u. 2, H-7623 Pecs, Hungary; E-Mails: orsolya.farkas.dr@gmail.com (O.F.); zsike23@gmail.com (E.K.); jozsef.pal@aok.pte.hu (J.P.); attila.schwarcz@aok.pte.hu (A.S.); tamas.doczi@aok.pte.hu (T.D.); 2saturn@gmail.com (A.B.); sator77@gmail.com (P.B.); 3 Department of Anatomy and Neurobiology, Virginia Commonwealth University, 1101 E. Marshall Street Richmond, Richmond, VA 23219, USA; E-Mail: jpovlish@hsc.vcu.edu; 4 Department of Surgery, Medical School, University of Pecs, Ret u. 2., H-7623 Pecs, Hungary; E-Mail: szantozalan@gmail.com

**Keywords:** endogenous, injury, neuroprotection, pituitary adenylate cyclase activating polypeptide, trauma

## Abstract

Pituitary adenylate cyclase activating polypeptide (PACAP) is a bioactive peptide with diverse effects in the nervous system. In addition to its more classic role as a neuromodulator, PACAP functions as a neurotrophic factor. Several neurotrophic factors have been shown to play an important role in the endogenous response following both cerebral ischemia and traumatic brain injury and to be effective when given exogenously. A number of studies have shown the neuroprotective effect of PACAP in different models of ischemia, neurodegenerative diseases and retinal degeneration. The aim of this review is to summarize the findings on the neuroprotective potential of PACAP in models of different traumatic nerve injuries. Expression of endogenous PACAP and its specific PAC1 receptor is elevated in different parts of the central and peripheral nervous system after traumatic injuries. Some experiments demonstrate the protective effect of exogenous PACAP treatment in different traumatic brain injury models, in facial nerve and optic nerve trauma. The upregulation of endogenous PACAP and its receptors and the protective effect of exogenous PACAP after different central and peripheral nerve injuries show the important function of PACAP in neuronal regeneration indicating that PACAP may also be a promising therapeutic agent in injuries of the nervous system.

## 1. Introduction

Pituitary adenylate cyclase activating polypeptide (PACAP) was isolated from ovine hypothalamus based on its ability to activate adenylate cyclase in the pituitary gland. PACAP exists in two forms: PACAP38 and PACAP27 with 38 and 27 amino acid residues, respectively [1]. PACAP38 is the predominant form in mammalian tissues. PACAP27 shares 68% sequence identity with vasoactive intestinal polypeptide (VIP), identifying PACAP as a member of the secretin/glucagon/growth hormone-releasing hormone (GHRH) superfamily [2]. Two types of PACAP binding sites have been characterized based on their relative affinities for VIP and PACAP. PAC1 receptor exhibits a high affinity for both PACAP forms and a low affinity for VIP, whereas VPAC1 and VPAC2 receptors bind both VIP and PACAP with high affinity. These receptors are members of G protein-coupled receptors (GPCR) family [3].

PACAP is a bioactive peptide with diverse activities in the nervous system. In addition to its more classic role as a neuromodulator, PACAP functions as a neurotrophic factor [4–7]. A number of studies have shown its neuroprotective effect *in vitro* and *in vivo* in different models of ischemia, neurodegenerative diseases and retinal degeneration [8–16].

Numerous neuroprotective drugs and therapeutic interventions have been tested in different animal models of neuronal injuries such as peripheral nerve lesions and traumatic brain injury models. Several neurotrophic factors, such as brain-derived neurotrophic factor, nerve growth factor and fibroblast growth factor, have been shown to play an important role in the endogenous response following both cerebral ischemia and traumatic brain injury, and to be effective when given exogenously [17–20]. Although cerebral ischemia and traumatic brain injury (TBI) have different pathogenesis, they share some common pathways including excitotoxicity, overproduction of free radicals, nitric oxid production, elevated Ca^2+^ level, and apoptosis [20–24]. Based on the several lines of evidence on the neurotrophic effect and neuroprotective effect of PACAP in global and focal cerebral ischemia [11,25–28], numerous experiments have been performed to investigate the effects of PACAP on traumatic nerve injuries. The aim of this review is to summarize the findings on the neuroprotective potential of PACAP in models of different traumatic nerve injuries.

### 1.1. Changes in the Expression of Endogenous PACAP and Its Receptors in Central Nervous System Injuries

PACAP is widely expressed in the embryonic brain at the onset of neurogenesis and it is strongly upregulated in several models of neuronal injuries [29]. Regeneration of the nervous system after injury is likely to require reemployment of mechanisms used to regulate brain development in the embryonic and postnatal periods. PACAP has very important role in the brain development, therefore, the expression of ligand and/or receptors might increase after various type of injury [29]. It is well known that the neurons in the cortex and hippocampus are particularly vulnerable in brain trauma [30]. The exact localization of PACAP mRNA expression in the developing and adult rat brain, and the presence of PACAP and PAC1 receptor mRNA are well described, suggesting important function of this peptide in these brain regions [31,32]. PACAP mRNA is expressed in alternating layers (I, III, and V layer) of the cerebral cortex, and it is localized to the CA1 and CA4 subregions of the hippocampus and the dentate gyrus, but it is not expressed in the CA2 or CA3 regions. PAC1 receptor mRNA is also highly expressed in the dentate gyrus and it has lower level in other parts of the hippocampus and the cortex [33]. Skoglosa *et al.* [33] examined changes in mRNA expression of PACAP and its type 1 receptor in the cortex and hippocampus after a moderate traumatic brain injury. This moderate trauma was performed by a 21 g free-falling weight that was dropped from a height of 35 cm on a piston. The traumatic injury produced an increase in the expression of PACAP mRNA within the ipsilateral cortex with most marked increases at 24 h to 72 h after the injury. PACAP mRNA expression was strongly increased in the perifocal area of the cortex around the lesion, but in the center of the injury the expression decreased. In the ipsilateral dentate gyrus PACAP mRNA expression was also increased with a peak at 12 h and 24 h after the injury. The hilar region of the hippocampus also expressed high levels of PACAP mRNA at 24 h after the trauma. In the naive animals there was a high expression of PAC1 receptor mRNA in the dentate gyrus, which showed a rapid, significant decrease on the ipsilateral side after the injury. The level of PAC1 receptor mRNA was at a minimum 6 h after the injury and it increased thereafter reaching control level at 72 h post-injury. In this experiment the authors described that the number of TUNEL-positive cells decreased in the cortex at 12 h after the injury when the expression of PACAP and PAC1 receptor mRNAs increased. This suggests that the increase in PACAP mRNA expression and the parallel decrease of apoptosis might be linked and PACAP could promote the survival of cortical neurons in TBI [33].

Van Landeghem *et al.* [34] examined the expression of PACAP immunoreactivity in human TBI. PACAP27 and PACAP38 immunoreactivities were significantly decreased in the immediate contusion regions after traumatic injury. The number of cells expressing PACAP27 and 38 was significantly increased in the pericontusional cortex showing the highest number within the first 7 days following TBI and maintained up to 99 days. The relative fraction of PACAP-positive reactive astrocytes was also increased, indicating the possibility of a complex endogenous neuroprotective mechanism exerted by PACAP after TBI [34].

In contrast to TBI models, no changes were observed in the cortical stab injury in rats, where a needle was inserted through the cortex into the underlying thalamus. Neither PACAP nor PAC1 receptor mRNA expression was upregulated in response to the glial hypertrophy and hyperplasia associated with the penetrating wound. There was no altered PACAP and PAC1 receptor mRNA expression in the lesion penumbra, callosal neurons in the contralateral cortex, or thalamic afferents [35]. The results of this stab injury model were in contrast with those reported by Skoglosa and coworkers [33] and maybe due to the differences between the two trauma models. It is known that the stab injury does not induce an inflammatory response as severe as the contusion model. The upregulation of PACAP in the compression contusion model may reflect an inflammatory response to the severe trauma. The stab injury penetrates the lateral ventricle and cerebrospinal fluid-derived factors could also attenuate PACAP expression [35]. While PAC1 receptor expression did not change PACAP may act on VIP receptors, and PACAP release may be increased in the absence of increased mRNA as in the case of other neuropeptides [35,36] (Table 1. I.).

PACAP38 immunopositive fibers have also been shown in the rat spinal cord and the medulla [37]. Numerous PACAP38 positive nerve fibers were detected in different layers of dorsal horn and the intermediate cell columns. In the medulla, immunoreactive fibers were observed for example in the spinal trigeminal tract and the solitary tract [37]. PACAP mRNA expression was also observed in neurons primarily in dorsal horn of the spinal cord and around the central canal. In addition, PACAP mRNA expression was detected in a few neurons in the ventral horn [38]. The important role of PACAP in spinal cord injury has been confirmed by another experiment, where VPAC1 receptor agonist suppressed the upregulation of inflammatory TNFα mRNA induced within 2 h after rat spinal cord injury [39]. Tsuchikawa and coworkers [40] have shown the neuroprotective effect of endogenous PACAP in a contusion model of spinal cord injury. In heterozygous PACAP knockout mice the recovery was delayed and the injury volume and number of injured neurons were significantly higher compared to wild-type mice.

### 1.2. Effect of PACAP Treatment in Central Nervous System Injuries

Recently, our research group described the protective effect of exogenous PACAP in different TBI models in the rat. Diffuse brain injury, particularly diffuse axonal injury (DAI) evoked by inertial forces provides a good model not complicated with abundant tissue laceration and a catastrophic activation of a wide range of proteolytic processes like in the case of more complex contusional/focal injuries [51,52]. Models of TBI, primarily or exclusively leading to DAI in well circumscribed brainstem pathways, like the Marmarou model [53,54] provide an excellent field of research as far as pathophysiology-driven therapeutic interventions are considered. In this model a simple weight-drop device is used consisting of a segmented brass weight free-falling through a Plexiglas guide tube. The animals are injured from 2 m with a 450 g weight and the skull fracture is prevented by cementing a small stainless-steel disc on the calvaria modeling impact acceleration head injury.

Using this model, our light microscopic examination showed that the vehicle- and drug-treated animals subjected to TBI and reacted for the visualization of β-amyloid precursor protein (APP) and RMO-14 antibodies revealed discrete focal immunoreactivity within scattered axons in the corticospinal tract (CSpT) and in the medial longitudinal fascicle (MLF). The polyclonal antiserum targeting the C-terminus of β-APP can be used for a marker of altered axoplasmic transport showing swollen, occasionally disconnected axon segments [55,56]. The RMO-14 antibody is known to exclusively target an epitope on the rod domains of altered neurofilament medium molecular weight (NF-M) subunits, which are exposed upon modification of NF sidearms, and is assumed to be the consequence of calcium induced enzymatic processes during TBI [57–59], the damaged axons are lobulated, vacuolated and partially or totally disconnected.

Pre-injury intravenous administration of 125 μg/kg PACAP did not alter the mean densities of β-APP-immunpositive axons in the corticospinal tract and medial longitudinal fascicle compared to control animals measured 2 and 6 h post-injury. However, when 100 μg PACAP was used intracerebroventricularly (icv.) immediately after the injury the number of β-APP-immunpositive axons was significantly reduced in the corticospinal tract 2 h after the injury compared to non-treated animals. Treatment with lower doses of PACAP (1 and 10 μg) did not reduce the axonal damage. In contrast to the corticospinal tract, no statistical significance could be observed between the various treated groups in the medial longitudinal fascicle [60].

In acute cerebrospinal injuries, it is of utmost importance to determine the therapeutic time window of neuroprotective agents, since the immediate therapy is not possible in most cases. Treatment with PACAP (100 μg) icv. 30 min or 1 h after TBI significantly reduced the β-APP-immunpositivity in the corticospinal tract 2 h after the injury compared to non-treated animals. There was no significant difference between the number of β-APP-immunpositive axons in the medial longitudinal fascicle and RMO-14 positive axon profiles in either tracts compared to control groups [61].

Kovesdi and coworkers [62] investigated the effect of 100 μg PACAP treatment 30 min after the impact acceleration TBI on the motor functions of rats. PACAP significantly improved the motor function from the third day compared with vehicle-treated animals in the beam-balance test, which is able to examine the vestibulomotor functions. In the elevated plus-maze test significant difference was also observed in the vehicle and PACAP-treated group on the sixth day after the trauma, because PACAP-treated animals spent less time in closed arm than vehicle-treated animals. These results indicate that PACAP is able to improve the trauma induced behavior changes.

Another frequently used model of diffuse TBI is the fluid percussion head injury model. The fluid percussion injury model evokes diffusely injured axons in the brainstem due to the shock waves evoked by fluid percussion rather than acceleration-deceleration as the Marmarou model does [53,63,64]. In this model icv. administration of 100 μg PACAP 30 min after the injury significantly reduced the density of β-APP- and RMO-14-immunopositive axon profiles in the corticospinal tract compared to control animals. In the medial longitudinal fascicle, no significant difference was observed between the density of β-APP- and RMO-14-immunopositive axons in PACAP versus vehicle-treated animals [65]. In these trauma models PACAP had no protective effect in medial longitudinal fascicle compared to corticospinal tract. This finding might be explained by the differences between the structures of fibers situated in the corticospinal tract and the medial longitudinal fascicle, and the different pathogenesis of axonal damage in the structures could also be the reason for different effects [66,67].

In a spinal cord injury model laminectomy is performed at T9-T10, and the dorsal surface of the spinal cord is compressed by dropping 10 g rod from a height of 25 mm (moderate injury) or 50 mm (severe injury) leading to massive neuronal cell death. In this model PACAP was injected 0.8–1.0 mm into the dorsal column of the spinal cord (0.5 or 1 μg PACAP in a case of moderate injury and 1 or 2 μg PACAP in severe injury). Eight days after the injury PACAP treatment significantly decreased the number of apoptotic cells and DNA fragmentation rostral and caudal to the lesion center compared to control animals. Two weeks later greater extended neuronal fibers with neurofilament (NF) immunostaining were found compared with untreated animals [68]. The delayed treatment of PACAP with human mesenchymal stem cells (hMSC) increased the remaining neuronal fibers in the injured spinal cord 7 days after the injury and these animals showed better locomotor functional recovery when compared to treatment with PACAP or hMSCs. The combined treatment also elevated the levels of antioxidant enzymes, Mn-superoxide dismutase and peroxiredoxin-1/6 to promote neuronal cell survival [69] (Table 2. I.).

### 1.3. Changes in the Expression of Endogenous PACAP and Its Receptors in Peripheral and Cranial Nerve Injuries

#### 1.3.1. Spinal Nerve Injury, Dorsal Root Ganglia

The examination of peripheral nerve injuries aims to find treatments to help in the neuronal regeneration for limiting the consequences of nerve injury [72]. The most commonly used peripheral nerve for experimental injury is the sciatic nerve. Transganglionic degeneration occurs after axotomy, with the loss of dorsal root ganglion (DRG) neurons and degeneration of their central projections in the dorsal horn [73]. Axotomy also induces changes of neuropeptide phenotype of primary sensory neurons. Moller *et al.* [74] showed that PACAP-immunopositive nerve cells with small diameter are found in the DRG and trigeminal ganglia in rats. With immunohistochemistry they demonstrated that PACAP-immunoreactive nerve fibers are present in the superficial layer of the dorsal horn of the spinal cord at the cervical, thoracic and also lumbar levels. Subsequently, the same research group provided more evidence for the occurrence of PACAP mRNA in DRG neurons and trigeminal ganglion with in situ hybridization [75]. The expression of PACAP in human primary sensory neurons has also been confirmed [76].

PACAP mRNA expression was also observed in neurons primarily in the dorsal horn of the spinal cord and around the central canal. In addition, PACAP mRNA expression was detected in a few neurons in the ventral horn [38]. The PACAP mRNA expression was elevated mostly in the larger neurons after sciatic nerve transection [38]. Zhang *et al.* [41,42] also examined the changes in PACAP level in the DRG after sciatic nerve injury. Axotomy of the sciatic nerve induced a rapid and prominent increase of PACAP and its mRNA levels after 15 h and peaked at 3 days after the injury, when 51% of the neurons showed PACAP immunoreactivity in contrast in normal conditions, where 17.5% of the neurons expressed PACAP. After the ligation of the sciatic nerve, accumulation of PACAP was mainly seen proximal to the injury but also distally, suggesting both anterograde and retrograde transport of the peptide [41]. Another similar study demonstrated that in the sciatic nerve stump PACAP27 and PACAP38 concentration increased after axotomy. In the spinal cord PACAP27 concentration also increased significantly after injury, but in contrast to the sciatic nerve stump PACAP38 level did not show significant changes [42]. Jongsma *et al.* [77] described elevated PACAP immunoreactivity in the gracile nucleus after sciatic nerve transection. The neurotrophins nerve growth factor (NGF) and neurotrophin 3 (NT-3) are important regulators in sensory neurons, and they modulate PACAP expression after L4-L6 spinal nerve injury. The intrathecal administration of NT-3 moderated the elevated PACAP expression in DRG neurons; in contrast, NGF significantly increased the PACAP expression in both intact and injured DRG neuron after axotomy [43]. These results suggest that PACAP could play a very important function in the protection of the neurons after peripheral nerve transections and it has a complex regulatory function.

The loose ligation of the sciatic nerve with chromic cat gut ligature is the chronic constriction injury (CCI) model of neuropathic pain [45]. Increased VPAC2 receptor mRNA expression was shown by in situ hybridisation within the ipsilateral dorsal horn following neuropathy, while VPAC1 receptor expression decreased and PAC1 receptors remained unchanged [45]. Electrophysiological studies showed that selective VPAC1, VPAC2 and PAC1 receptor antagonists inhibited mustar oil- and attenuated cold-induced neuronal activity in CCI model, but not brush-induced activity of the dorsal horn neurons [45].

Another model for sciatic nerve injury is the use of a narrow silicone tube which is applied around the nerve and it is compressed for various time periods. Pettersson and coworkers [44] detected significant elevation in the number and density of PACAP mRNA expression in DRG, and also found increased number of PACAP-immunoreactivity in DRG neurons and in the compressed sciatic nerve segment (Table 1. II.1.).

#### 1.3.2. Autonomic Nerve Injury, Autonomic Ganglia

PACAP and VIP are present in autonomic parasympathetic neurons, such as otic and sphenopalatine ganglia and in the jugular-nodose ganglion, which is a mixed parasympathetic/sensory ganglion in rats and also in humans [78–80]. On the other hand, PACAP has important roles also in the sympathetic transmission, because it induces catecholamine and NPY synthesis and release mediated by PAC1 receptors on superior cervical ganglion [81]. PACAP immunoreactivity is present in the preganglionic nerve fibers and in few neurons of the superior cervical ganglion and PAC1 receptors occur in all nerve cell bodies [46]. After the postganglionic transection of the external and internal carotid nerves, increased PACAP and decreased PAC1 receptor expression was observed in the superior cervical ganglion. Chemical sympathectomy induced by 6-hydroxydopamine led to similar changes. In contrast, preganglionic denervation showed limited effect on the expression of PACAP and there was no effect on the expression of PAC1 receptors [46]. It is known that some superior cervical ganglion neurons project their axons back into the sympathetic chain, therefore, the authors assume that the increased PACAP expression after preganglionic denervation was due to the axotomy of these neurons rather than the loss of preganglionic innervation [46]. After the transection of the cervical sympathetic trunk PACAP-immunoreactive nerve endings on postganglionic neurons that mainly were of preganglionic origin disappeared in the superior cervical ganglia [47] (Table 1. II.2.).

#### 1.3.3. Cranial Nerve Injuries

The facial nerve innervates the muscles of facial expression and it can be easily damaged due to its anatomical characteristics. Facial nerve axotomy is another frequently utilized model to study motorneuron degeneration and regeneration to test the effect of different trophic factors. Axotomy of the facial nerve induces a rapid response in the ipsilateral facial motor nucleus involving motor neurons, astroglia and microglia [82]. Zhou *et al.* [48] investigated the changes in PACAP, PAC1 and VPAC2 receptor mRNA expression in the facial motor neurons after facial nerve axotomy. PACAP gene expression was very low in contrast to PAC1 and VPAC2 receptors which showed high expression in facial motoneurons of normal rats. Six hours after the axotomy a robust time-dependent increase in PACAP mRNA was observed in the facial motor nucleus, which peaked at 48 h, but remained elevated 30 days after the injury compared to the contralateral side. In contrast to PACAP, PAC1 receptor gene expression significantly decreased, but VPAC2 mRNA expression did not change in facial motor neurons after axotomy indicating the dominancy of VPAC2 receptor signaling pathways in this damage. Armstrong *et al.* [83] demonstrated that this induction of PACAP mRNA after the axotomy requires inflammatory mediators using severe combined immunodeficiency (SCID) mice, but leukemia inhibitory factor (LIF), interleukin-6 (IL-6) and tumor necrosis factor α (TNFα) are not required for this response to injury [83]. PACAP-deficient mice are used to investigate actions of endogenous PACAP after facial nerve injury. After axotomy there was no significant difference in the motor neuron survival between wild type and PACAP-deficient mice, but after crush injury the axon regeneration in PACAP-deficient mice was significantly delayed [49], suggesting that PACAP might also act in the process of axonal regeneration after injury. The impaired regeneration was associated with significantly increased levels of proinflammatory cytokine (TNFα, interferon γ, IL-6) and decreased levels of anti-inflammatory gene expression (IL-4) in both the facial motor nucleus and nerve crush site. Further investigations are necessary to describe the exact relationship between the inflammatory changes and the impaired nerve regeneration in PACAP-deficient mice [49].

PACAP mRNA expression increased in the ipsilateral mesencephalic trigeminal nucleus 3 h after the axotomy of the main trunk of the masseteric nerve and peaked 24 h after the surgery. There was an increase in PACAP38-immunoreactivity by radioimmunoassay analysis in the nerve proximal to the transection compared to the uninjured side [50] (Table 1. II.3.).

### 1.4. Effect of PACAP Treatment in Cranial Nerve Injuries

There are limiting experiments investigating the effect of exogenous PACAP treatment in different cranial nerve injuries. The possible protective effect of PACAP in axotomy is supported by a study, where 100 nM PACAP injected at the transected sites after the transection of the facial nerve improved neuromuscular recovery. In this study PACAP facilitated the recovery of compound muscle action potential increasing the number of regenerating myelinated axons and increased the neurotrophin GDNF expression in orbicularis oris muscle [70]. Although PACAP as a neurotrophic factor helped in the regeneration of the axons and impaired the functional outcome of behavioral tests, it stimulated the growth of the collateral axon branches limiting its utilization in the clinical treatment [84].

It is known that PACAP has neuroprotective effects in the retina in different pathological conditions [14]. Optic nerve injury leads to optic neuropathy, which is similar to the glaucoma-induced neuronal damage. Fourteen days after optic nerve transection the number of retinal ganglion cells decreased compared to the uninjured side, but intravitreal PACAP treatment (10 or 100 pM) significantly improved the retinal ganglionic cell survival compared to the control group [71] (Table 2. II.).

## 2. Conclusions and Perspectives

Traumatic brain injury is a major health care problem, representing the primary cause of mortality and severe disability in the first four decades and expected to become the third most frequent cause of death until 2020 worldwide [85,86]. Despite all efforts and scientific programs facilitating a bench to bedside approach, so far none of the therapeutic treatments and agents that have worked at the preclinical setting proved its efficacy in the clinical treatment of the head injured [87]. These disappointing results can be explained by the complexity of injury as well as that of the injured organ and also by a failure to identify common endpoints that may couple preclinical and clinical findings [88,89].

The upregulation of endogenous PACAP and its receptors and the protective effect of exogenous PACAP after different central and peripheral nerve injury show the important function of PACAP in the neuronal regeneration. The results of the summarized experiments indicate that PACAP may also be a promising therapeutic agent in the injuries of the nervous system. Many therapeutic agents have been used in models of traumatic nerve injuries, such as antiapoptotic and antiinflammatory drugs, to attenuate the main pathogenetic mechanism present in nervous injuries [20]. PACAP has a well-known antiapoptotic effect against various agents in different neuronal and non-neuronal cell lines [5,90–95]. Current thought appreciates that DAI is associated with membrane perturbation caused by acceleration-deceleration type TBI leading to an influx to Ca^2+^ activating proteolytic processes and Ca^2+^ sequestration in mitochondria [96]. Initially pathobiological processes are predominantly of necrotic nature but Ca^2+^-pooling and proapoptotic signals will lead to the activation of mitochondrial permeability transition with massive release of cytochrome-c (cyto-c), apoptosis activating factor-1 (APAF-1) and other proapoptotic substances leading to the activation of caspase-dependent apoptotic cell death/axonal demise [23,51,97–100]. Aconitase, a key mitochondrial enzyme influencing the viability of neurons in response to oxidative stress, is inactivated by a deprivation of Ca^2+^ influx into neurons and PACAP attenuates this inactivation [101]. PACAP also reduces cytochrome c release from the mitochondria and caspase-3 activation, which is also activated in traumatic brain injury [23,102].

Inflammation is not restricted to infectious or autoimmune disorders of the nervous system, but occurs in cerebral ischemia, trauma and neurodegenerative disorders [20]. PACAP has been proven to be a potent inactivator of induced microglial release of proinflammatory cytokines and chemokines such as TNFα, IL-1β, IL-6, IL-12 and NO [39,103,104]. Moreover, PACAP stimulates the anti-inflammatory cytokine IL-10 [105]. Numerous studies have demonstrated that the anti-inflammatory actions of PACAP exists also *in vivo* [106].

One of the lessons learned from the failure of recent trials and translational research projects is that therapeutic strategies should most probably be based on a “polypharmacia”-like approach, not excluding “dirty drugs” with multiple targets [51,88,107–109].

Approaches simultaneously targeting apoptotic and necrotic processes harbor particular therapeutic potential. To this end, the line of evidence pointing to the therapeutic efficacy of PACAP in various models of TBI mandates further exploration.

## Figures and Tables

**Table 1:** Changes in the expression of endogenous pituitary adenylate cyclase activating polypeptide (PACAP) and its receptors in central and peripheral nervous system injury.

I. Changes in the expression of endogenous PACAP and its receptors in central nervous system injury				
Type of the injury	Changes in PACAP and its receptors	Examined region	Species	References
moderate traumatic brain injury	PACAP mRNA↑	ipsilateral perifocal lesion (cortex), gyrus dentatus	rat	[33]
	PAC1 receptor mRNA↓	gyrus dentatus		
traumatic brain injury	PACAP27↓; PACAP38↓	traumatized neocortex	human	[34]
	PACAP27↑; PACAP38↑	pericontusional neocortex		
cortical stab injury	PACAP mRNA no change	lesion penumbra, callosal neurons in the contralateral cortex, and thalamic afferents	rat	[35]
	PAC1 receptor mRNA no change			
**II. Changes in the expression of endogenous PACAP and its receptors in peripheral and cranial nerve injuries**				
**1. Spinal nerve injury**				
sciatic nerve transection	PACAP38↑; PACAP mRNA↑	dorsal root ganglion	rat	[41–43]
	PACAP27↑; PACAP38 no change	spinal cord		[42]
	PACAP27↑; PACAP38↑	sciatic nerve		
	PACAP mRNA↑	ventral horn of spinal cord	rat	[38]
sciatic nerve compression	PACAP↑; PACAP mRNA↑	dorsal root ganglion, sciatic nerve	rat	[44]
chronic constriction injury of sciatic nerve	PAC1 receptor mRNA no change;VPAC1 receptor mRNA↓; VPAC2 receptor mRNA↑	ipsilateral dorsal horn of spinal cord	rat	[45]
**2. Autonomic nerve injury**				
postganglionic transection of external and internal carotid nerves	PACAP↑; PAC1 receptor↓	superior cervical ganglion	rat	[46]
6-hydroxydopamineinduced chemical sympathectomy				
preganglionic denervation of sympathetic chain	PACAP↑; PAC1 receptor no change			
cervical sympathetic trunk transection	PACAP↓	preganglionic nerve fibers of superior cervical ganglion	rat	[47]
**3. Cranial nerve injury**				
facial nerve transection	PACAP↑; PAC1 receptor↓; VPAC2 receptor mRNA no change	facial motor nucleus	rat	[48]
		axon regeneration delayed	PACAP deficientmouse	[49]
masseteric nerve transection	PACAP mRNA↑	ipsilateral mesencephalic trigeminal nucleus	rat	[50]
	PACAP38↑	masseteric nerve proximal to the transection		

**Table 2:** Effect of PACAP treatment in central and peripheral nervous system injury.

I. Effect of PACAP treatment in central nervous system injury				
Type of injury	Treatment	Effect of treatment	Species	References
Marmarou model of traumatic brain injury	icv. 100 μg PACAP 0 min, 30 min or 1 h postinjury	β-APP immunopositivity in CSpT↓, in MLF no change	rat	[60,61]
		RMO-14 immunopositivity in CSpT and in MLF no change		
fluid percussion injury	icv. 100 μg PACAP postinjury	β-APP immunopositivity in CSpT↓, in MLF no change	rat	[65]
		RMO-14 immunopositivity in CSpT↓ and in MLF no change		
spinal cord injury	0.5–2 μg PACAP into dorsal column	apoptotic cell number and DNS fragmentation↓ rostral and caudal to the lesion center	rat	[68]
	2 μg PACAP + 2 × 10^5^ hMSCs into dorsal column	better locomotor function; antioxidant enzymes↑		[69]
**II. Effect of PACAP treatment in cranial nerve injuries**				
facial nerve injury	100 nM PACAP injected at transected side	neuromuscular recovery ↑	rat	[70]
optic nerve transection	intravitreal 10–100 pM PACAP	retinal ganglionic cell survival↑	rat	[71]
